# Assessment of Industrial Antimony Exposure and Immunologic Function for Workers in Taiwan

**DOI:** 10.3390/ijerph14070689

**Published:** 2017-06-26

**Authors:** Chin-Ching Wu, Yi-Chun Chen

**Affiliations:** 1 Department of Public Health, China Medical University, 91 Hsueh-Shih Road, Taichung 404, Taiwan; wucc@mail.cmu.edu.tw; 2 Department of Health Management, College of Medicine, I-Shou University, 8 Yida Road, Yanchao District, Kaohsiung 824, Taiwan

**Keywords:** antimony, antimony trioxide, biomarker, engineering plastics, glass, immunologic function, IgG, IgA, IgE

## Abstract

This study investigated antimony exposure among employees in industries in Taiwan and evaluated whether their immunologic markers were associated with antimony exposure. We recruited 91 male workers and 42 male office administrators from 2 glass manufacturing plants, 1 antimony trioxide manufacturing plants, and 2 engineering plastic manufacturing plants. Air samples were collected at worksites and administrative offices, and each participant provided specimens of urine, blood, and hair to assay antimony levels. We also determined white blood cells, lymphocyte, and monocyte, IgA, IgE, and IgG in blood specimens. The mean antimony concentration in the air measured at worksites was much higher in the antimony trioxide plant (2.51 ± 0.57 mg/m^3^) than in plastic plants (0.21 ± 0.06 mg/m^3^) and glass plants (0.14 ± 0.01 mg/m^3^). Antimony levels in blood, urine, and hair measured for participants were correlated with worksites and were higher in workers than in administrators. The mean serum IgG, IgA, and IgE levels were lower in workers than in administrators (*p* < 0.001). Serum IgA and IgE levels in participants were negatively associated with antimony levels in air samples of workplaces, and in blood, urine, and hairs of participants. Serum IgG and IgE of all participants were also negatively associated with antimony levels in their hairs. In conclusion, the antimony exposure is greater for workers employed in the five industrial plants than for administrators. This study suggests serum IgG, IgA, and IgE levels are negatively associated with antimony exposure.

## 1. Introduction

Antimony (Sb) and its compounds are widely used in some industries, including alloy, plastic, glass, and textile industries [1,2,3,4,5,6]. The permissible exposure limit (PEL) established by the US Occupational Safety and Health Administration for exposure to antimony and compounds is 0.5 mg/m^3^ of antimony in the workplace based on a working 8 h shift and a 40 h week [7]. Taiwan has also adopted the 8 h time weighted average concentration of antimony and its compounds in the air of workplace at a level of 0.5 mg/m^3^ [8]. Antimony and its compounds may irritate eyes, skin, and respiratory systems, and are suspected to be carcinogenic and teratogenic substances [1,9,10,11,12,13]. In an animal study, among rats exposed to substances containing Sb, only female rats developed lung cancer; 27% in those rats exposed to antimony trioxide and 25% in those rats exposed to antimony ore had the cancer [10]. Grosskopf et al. reported that trivalent antimony was responsible for genotoxicity in the cellular system because antimony could partly impair the pathway of nucleotide repair [11]. DNA damage has been detected for workers with occupational exposure to antimony trioxide [12,13]. International Agency for Research on Cancer (IARC) has classified trivalent antimony as a possible human carcinogen [14]. 

Studies investigating the impact of antimony exposure on the human immune system are limited. A survey on occupational contact dermatitis and sensitization among 126 workers employed in the ceramics industry showed that 48 (25.3%) workers were found to be sensitized to various exposures. Among them, two persons were sensitized to antimony trioxide [1]. Huang et al. found that urinary levels of metals, including antimony, were higher in patients with asthma than subjects without asthma [15]. In an earlier study in workers at an antimony trioxide manufacturing plant, Kim et al. found the serum immunoglobulin levels, such as IgG1 and IgE, were lower in workers exposed to antimony than in controls [2].

In 2010, Taiwan imported 5501 tons of antimony trioxide and produced 14,100 tons of the chemical. Workers in the antimony industry are likely exposed to a large amount of antimony than general population, particularly for workers exposed to antimony trioxide at the nanoparticle size of 0.6 µm emitted with nano-technology. This study investigated antimony exposure levels among workers and administrative staff employed in three types of industry associated with manufacturing antimony compounds or using them, including industries producing antimony trioxide, and industries using antimony for manufacturing glass and engineering plastic products. In the glass manufacturing industry, sodium antimonite is used to decolorize and refine glass [5]. Antimony trioxide is also used as a polycondensation catalyst in the plastic manufacturing industry to synthesize polyethylene terephthalate [5]. Workers at these three types of industry may be exposed to various levels of antimony. We attempted to investigated whether the immune factors were associated with the antimony exposure levels among these workers. Administrative staff served as general population controls.

## 2. Materials and Methods

### 2.1. Study Groups

This study was conducted at one antimony trioxide manufacturing plant, two glass manufacturing plants, and two engineering plastic manufacturing plants, after obtaining the approval from the Research Ethics Committee at China Medical University & Hospital (DMR99-IRB-142(FR)). Ninety-one male workers were recruited at worksites as the metal exposure group, and 42 male administrators from these five plants as controls. With consent, we collected air samples at worksites and administrative offices at the 5 plants. Each participant provided three tubes of blood samples in the mornings: one 3 mL blood sample in a purple head tube for metal analysis, one 3 mL blood sample in a purple head tube for white blood cell (WBC) count, and a 10 mL blood sample in a red head tube for an immunoglobulin assay. We also collected urine and hair samples from participants to assess metal concentrations. All samples were shipped to laboratories at 4 °C. Samples for white blood cell counts and the immunoglobulin assay were sent to the hospital for analyses in 24 h. Blank samples were prepared on sites for all types of sample. Samples for metal tests were stored at −70 °C until analysis. We used Inductively Coupled Plasma Mass Spectrometer (ICP-MS) (Perkin-Elmer SCIEX ELAN DRC II, Concord, ON, Canada) to determine the antimony concentrations in the blanks, samples, and standards (ICP multi element Standard Solution, Merck, Darmstadt, Germany). The limit of detection (LOD) and the limit of quantification (LOQ) were performed. The spike recovery test or reference materials recovery test was performed as well. Analyzers were blinded to sample identifications. A questionnaire was used to collect information on personal characteristics, including birth date, work histories, lifestyle (i.e., smoking, drinking, betel nut chewing, etc.), and physician diagnosed allergy status.

### 2.2. Sample Collection and Analysis for Antimony

#### 2.2.1. Air Sample

The environmental air sampling devices were set up at a 120 cm height at worksites and administrative offices to collect the particulates in the air using a 37 mm filter cartridge containing a 0.5 μm PolyVinyl Chloride (PVC) filter. Using a flow rate of 2 L/min, personal samplers (Gillian Instrument Corp., West Caldwell, NJ, USA) were set for the sampling time from 5 to 7 h during work. Filter blanks and field blanks were prepared for quality control and were analyzed together with the samples. The filter in the sampling tube was treated with a mixture of 5 mL of 37% hydrochloric acid and 1 mL of 70% nitric acid, followed by ultrasonic shock for 30 min, and filtered using a Milipore filter membrane with a 0.22 μm pore size. The filtrate was diluted with 1% (*v*/*v*) of hydrochloric acid and nitric acid [16]. We used ICP-MS (Perkin-Elmer SCIEX ELAN DRC II, Waltham, MA, USA) to determine the antimony concentration in the blanks, samples, and standards (ICP multi element Standard Solution, Merck, Darmstadt, Germany). LOD and LOQ were 4.4 µg and 15 µg, respectively. The spike recovery test of antimony was 84.5%.

#### 2.2.2. Blood Sample 

For analyzing antimony in the blood, a 0.5 mL blood sample was placed into a vial with 3 mL of 70% nitric acid and digested using microwave. We took 1 mL of digested solution, added 1 mL of Indium standard solution (as an internal standard), mixed it with 8 mL of 1% (*v*/*v*) hydrochloric acid to make a 10 mL solution, and quantified blood antimony using ICP-MS (Perkin-Elmer SCIEX ELAN DRC II, Waltham, MA, USA) [17]. LOD and LOQ for the blood antimony determination were 0.06 µg/L and 0.12 µg/L, respectively. Seronorm Trace Elements Blood L-3 (ref. 102405) (Seronorm Pharmaca, Billingstad, Norway) was used as a reference material, and the recovery rate was 90.0%.

#### 2.2.3. Urine Sample

A clean glass vial was sent to each participant in advance. Each participant provided a vial of the first void urine specimen in the morning. We took a 1 mL urinary sample to measure creatinine. The remaining urinary samples were stored at −70 °C until ready for analysis. At room temperature, 10 mL of urinary samples were centrifuged at 3000 rpm for 5 min. We took 1 mL of upper solution mixed with 1 mL of Indium standard solution (as an internal standard) and 8 mL of 1% (*v*/*v*) of hydrochloric acid and nitric acid and then quantified urinary antimony using ICP-MS (Perkin-Elmer SCIEX ELAN DRC II, Waltham, MA, USA) [18]. The levels of urine antimony were adjusted for urinary creatinine (cre.) and expressed as ug/g cre. The LOD and LOQ for the urine antimony determination were 0.03 µg/L and 0.12 µg/L, respectively. Seronorm Trace Elements Urine L-2 (ref. 201205) (Seronorm Pharmaca, Billingstad, Norway) was used as a reference material, and the recovery rate was 90.2%.

#### 2.2.4. Hair Sample

A pinch of hairs near neck cut from each participant was collected in a sealed bag for analyzing the antimony concentration. We took 0.4 g of hairs and washed it twice with 1:200 (*v*/*v*) Triton X-100 solutions, followed by acetone, and finally washed twice with deionized water. The washed hairs were dried in an oven at 75 °C for 24 h, and then stored in an electronic dry cabinet (AD-51, EDRY Enterprise Co, Taipei, Taiwan) at room temperature for 12 h or longer until digestion. We measured 0.2 g of dried hairs in a vial with 3 mL of 70% nitric acid and digested using microwave. The digested hair solution was diluted to 10 mL with 1% (*v*/*v*) hydrochloric acid. We took 1 mL solution, mixed with 1 mL of Indium standard solution (as an internal standard) and 8 mL of 1% (*v*/*v*) hydrochloric acid and quantified hair antimony using ICP-MS (Perkin-Elmer SCIEX ELAN DRC II, Waltham, MA, USA) [19]. The LOD and LOQ for the hair antimony determination were 0.0004 µg/g and 0.012 µg/g, respectively. Certified reference hair (CRM GBW-09101-Human Hair, Shanghai Institute of Nuclear Research Academia Sinica, Shanghai, China) was used as a reference material, and the recovery rate was 81.0%.

### 2.3. White Blood Cell and Immunoglobulins Determination

The white blood cell counts were performed using flow cytometry (Automated Hematology Analyzer of Beckman Coulter LH series). The 10 mL blood sample was centrifuged to obtain serum for the measurement of IgA, IgG, and IgE. Serum IgA and IgG were measured using turbidimetry (Nephlometer, Hitachi 747, Tokyo, Japan), and serum IgE was quantified by Enzyme-linked immunoassay [20].

### 2.4. Statistical Analysis

Data analysis first compared the personal characteristics between all operation workers and all administrative staff recruited at the 5 plants. Distributions of age, employment history, lifestyles, and allergy history were examined using chi-square. Average antimony concentrations in air, blood, urine, and hair samples were compared between workers and administrative staff by industry type. Differences were examined using the Kruskal–Wallis test because antimony concentrations in the air samples among the 5 plants, and in the blood, urine, and hair samples of the participants, were not normally distributed. Counts of WBC, lymphocyte, monocyte, IgA, IgG, and IgE were stratified into 2 or 3 levels based on the range of reference values, and compared between all workers and all administrative staff, examined using chi-square. We also calculated and compared means of serum WBC, lymphocyte, monocyte, IgA, IgG, and IgE between workers and administrative staff using the Kruskal–Wallis test. The Spearman’s correlation coefficients, ρ (rho), were calculated between levels of antimony and of immunological indicators for all participants. IBM SPSS Statistics version 18 software (IBM Corp., Armonk, NY, USA) was used for data analyses, and the *p*-value was set at 0.05 as significant.

## 3. Results

### 3.1. The Attributes of Subjects

Table 1 shows that workers were younger and had shorter employment history than were administrators. However, the prevalence rates of smoking, drinking, betel nut chewing, and allergic history of metal-exposed workers and administrative staff were alike. Near 30% of study subjects smoked, used alcohol, and chewed betel nuts, and 21.8% of them had been diagnosed with an allergic disorder.

### 3.2. The Antimony Levels in the Air of Worksite and in Blood, Urine and Hairs Samples

Table 2 shows antimony levels in samples of air, blood, urine, and hair by industry type for workers and administrative staff. The mean antimony concentration in air samples measured for the antimony trioxide manufacturing plant was the highest (2.51 ± 0.57 mg/m^3^), near 18-fold higher than that for glass plants or 12-fold higher than that for engineering plastic manufacturing plants. The antimony concentrations in blood, urine, and hair measured for workers of antimony trioxide manufacturing plant were also the highest, at levels of 3.88 ± 1.10 μg/L, 27.15 ± 6.00 μg/g cre., and 0.10 ± 0.01 μg/g, respectively. The Spearman’s correlation analysis showed that antimony concentrations in blood, urine, and hair of participants were significantly associated with the concentrations in air samples with coefficients of 0.713, 0.870, and 0.865 (*p* < 0.01), respectively (data not shown). The measured antimony levels in air and in blood, urine, and hair samples were much lower for all administrative staff than for all workers (all *p* < 0.01).

### 3.3. White Blood Cell Count and Immunoglobulin Indicators 

Immunoglobulin levels of most participants in this study were in normal physiological reference ranges (Table 3). However, 9.0% of participants had the WBC levels higher than the reference values, and 24.1% of participants had lymphocyte levels below the reference values. The monocyte levels, IgA and IgE of workers and staff were in normal reference value ranges. However, the mean serum IgG, IgA, and IgE levels among workers were lower than that among administrative staff (*p* ≤ 0.001).

### 3.4. The Correlation between Immunological Levels and Antimony Levels

Table 4 shows correlations between immunological indicators of all participants and antimony levels in air, blood, urine, and hair samples. WBC levels had a positive relationship with antimony exposures, but not significant. The monocyte levels were negatively correlated with antimony levels in blood and urine, with the corresponding coefficients of −0.300 and −0.175 (*p* < 0.05), respectively. The serum IgG levels were negatively correlated with antimony levels in air samples at worksites and in hairs of participants (*p* < 0.05). The serum IgA and IgE levels also had significant negative correlations with antimony levels in air and in blood, urine, and hair. The Spearman’s ρ (rho) values were stronger for IgA, with coefficients of −0.366, −0.291, −0.355 and −0.370 (*p* < 0.001), associated with antimony levels in air, and in blood, urine, and hair, respectively.

## 4. Discussion

This study surveyed the antimony exposure levels for workers and administrative staff at manufacturing plants with antimony exposures and evaluated relationships between levels of antimony exposure and immunologic characteristics of participants. We surveyed glass, antimony trioxide, and engineering plastics manufacturing plants and found that the environmental antimony concentration in the air samples collected at these five worksites was the highest at the antimony trioxide manufacturing plant, more than five times the legal limit (PEL) of 0.5 mg/m^3^ of Taiwan. The antimony levels in blood, urine, and hair were also the highest in samples from workers of the antimony trioxide manufacturing plant, in response to the exposure from the air.

In this study, the antimony measured in blood, urine, and hair for participants were strongly associated with the antimony concentrations in the air to which they were exposed to. Our further data analysis showed that the relationship was stronger for levels in urine and in hair (coefficients of 0.870 and 0.865, respectively) than for levels in blood (a coefficient of 0.713) (data not shown). Antimony in urine and in hairs could be appropriate biomarkers for evaluating the exposure of antimony at worksites. However, in an occupational survey for antimony exposure in textile factory, Iavicoli et al. found that the air antimony levels of personal exposure ranged from 0.01 to 0.55 µg Sb/m^3^ and that the mean urinary antimony level of workers was 0.35 ± 0.29 µg Sb/L [3]. They considered the correlation between low environmental exposure and human burden is negligible. In an earlier survey at a lead battery factory, Kentner et al. found that the mean antimony levels in the air were 4.5 µg Sb/m^3^ in the grid casting area and 12.4 µg Sb/m^3^ in the lead plate stibine formation area [21]. The corresponding mean urinary levels in workers at the end of a week of exposure were 3.9 and 15.2 µg Sb/g creatinine, respectively. Their air and urinary antimony levels were greater than those we found in our study at the antimony plants. Lüdersdorf et al. assessed trivalent antimony exposure among glass refining workers and found that the urinary antimony levels were associated with the concentrations in the air samples of the worksites. This suggested that urinary antimony levels were useful in monitoring the exposure of antimony in work places [22].

Metals and organic chemicals have been associated with immunity [23,24,25,26]. Fewer studies have investigated the immunomodulatory associated with antimony exposure. We found that the serum IgG, IgA, and IgE levels were significantly lower among workers than among administrative staff and were negatively correlated with the antimony levels in the worksite air, and in the blood, urine, and hair of study participants. Our results are consistent with findings of an earlier study [2]: Kim et al. also found that the antimony exposure had an association with lower serum IgG1 and IgE levels [2]. However, we did not evaluate the relationship between the subclasses of IgG1 and antimony exposure. Immunoglobulins play an important role in anti-infection and in lowering the chance of cancer [23,24]. Whether the serum levels of IgG, IgA, and IgE suppressed in workers exposed to antimony increase the risk of infections or chronic disorders deserves further study.

This is one of the few studies exploring the correlation between antimony exposure and immunoglobulin levels, but has some limitations. The causal relationship between antimony exposure and immunological indicators cannot be established in this study because of its cross-sectional design. However, the antimony levels in hairs represent a historical exposure; there could be a negative relationship between antimony levels in the hair and serum levels of IgG, IgA, and IgE. Levels of neutrophils and eosinophils in white blood cells were not measured in this study, we were unable to measure whether levels of neutrophils and eosinophils are associated with the antimony exposure. Our sample size was not large enough to analyze these associations by age stratum or by work history.

## 5. Conclusions

The antimony levels in blood, urine, and hair were useful in evaluating the antimony exposure from worksites. Our study demonstrated that the high heterogeneity in antimony exposures from the air of five plants provided clear Spearman’s correlations with human immunity markers. The serum levels of IgG, IgA, and IgE were lower among workers exposed to antimony than among administrative staff and were negatively associated with antimony levels in hair. Whether the suppression of serum levels of IgG, IgA, and IgE associated with antimony exposure is detrimental to health deserves study.

## Figures and Tables

**Table 1:** Demographic and lifestyle characteristics of workers exposed to antimony and administrative staff.

Variables	Workers	Administrators	Total	*p*-Value *
	*N* = 91	*N* = 42	*N* = 133	
Age, years	*n* (%)	*n* (%)	*n* (%)	
<30	16 (17.6)	0	16 (12.0)	<0.001
30–39	30 (33.0)	9 (21.4)	39 (29.3)	
40–49	31 (34.1)	19 (45.3)	50 (37.6)	
50–59	14 (15.3)	8 (19.1)	22 (16.5)	
≥60	0	6 (14.2)	6 (4.5)	
Years at work				
<10	19 (20.9)	4 (9.5)	23 (17.3)	0.001
10~19	50 (55.0)	18 (42.9)	68 (51.1)	
20~29	22 (24.1)	15 (35.7)	37 (27.8)	
≥30	0	5 (11.9)	5 (3.8)	
Smoking				
Yes	26 (28.6)	13 (31.9)	39 (29.3)	0.78
No	65 (71.4)	29 (69.1)	94 (70.7)	
Drinking				
Yes	30 (33.0)	14 (33.3)	44 (33.1)	0.97
No	61 (67.0)	28 (66.7)	89 (66.9)	
Betel nut use				
Yes	28 (30.8)	12 (28.6)	40 (30.1)	0.80
No	63 (69.2)	30 (71.4)	93 (69.9)	
Diagnosed allergy				
Yes	17 (18.7)	12 (28.6)	29 (21.8)	0.20
No	74 (81.3)	30 (71.4)	104 (78.2)	

* Chi-square test.

**Table 2:** Average antimony concentrations in samples of air, blood, urine, and hair of metal-exposed workers and administrative staff by type of industry.

Factory	Antimony Concentration			
	Air (mg/m^3^)	Blood (μg/L)	Urine (μg/g cre. ^a^)	Hair (μg/g)
**Glass**				
Workers (*n* = 55)	0.14 ± 0.01	0.78 ± 0.21	5.60 ± 1.24	0.10 ± 0.01
Administrativestaff (*n* = 20)	0.007 ± 0.001	0.60 ± 0.11	2.55 ± 0.71	0.06 ± 0.01
*p*-value *	<0.001	<0.001	<0.001	<0.001
**Antimony trioxide**				
Workers (*n* = 14)	2.51 ± 0.57	3.88 ± 1.10	27.15 ± 6.00	5.66 ± 3.66
Administrativestaff (*n* = 9)	0.04 ± 0.01	1.07 ± 0.87	2.09 ± 0.55	0.04 ± 0.004
*p*-value *	<0.001	<0.001	0.001	<0.001
**Engineering plastic**				
Workers (*n* = 22)	0.21 ± 0.06	2.17 ± 0.48	7.48 ± 1.30	0.32 ± 0.05
Administrativestaff (*n* = 13)	0.004 ± 0.001	0.49 ± 0.05	1.86 ± 0.55	0.04 ± 0.004
*p*-value *	<0.001	<0.001	<0.001	<0.001
**Total**				
Workers (*n* = 91)	0.52 ± 0.88	1.61 ± 1.25	9.28 ± 6.31	1.00 ± 2.35
Administrativestaff (*n* = 42)	0.012 ± 0.015	0.602 ± 0.0.140	2.26 ± 0.0.68	0.048 ± 0.041
*p*-value *	<0.001	<0.001	<0.001	<0.001

* Kruskal–Wallis test. ^a^ cre.: creatinine.

**Table 3:** Distributions of levels of white blood cell and immunological indicators compared between workers and administrative staff.

Immunological Indicators	Workers	Administrators	Total	*p*-Value
	*N* = 91	*N* = 42	*N* = 133	
WBC, 10^3^/μL	*n* (%)	*n* (%)	*n* (%)	
<4	1 (1.1)	0	1 (0.8)	0.69
4–10 ^a^	81 (89.0)	39 (92.9)	120 (90.2)	
>10	9 (9.9)	3 (7.1)	12 (9.0)	
Mean (SD)	6.59 (1.99)	6.00 (1.71)	6.41 (1.92)	0.051
Lymphocyte, %				
<30	26 (28.6)	6 (14.3)	32 (24.1)	0.20
30–40 ^a^	61 (67.0)	34 (81.0)	95 (71.4)	
>40	4 (4.4)	2 (4.7)	6 (4.5)	
Mean (SD)	32.3 (4.78)	33.2 (3.99)	32.6 (4.56)	0.160
Monocyte, %				
<4	0	0	0	-
4–10 ^a^	91 (100.0)	42 (100.0)	133 (100.0)	
>10	0	0	0	
Mean (SD)	6.71 (0.76)	6.80 (0.82)	6.74 (0.78)	0.899
IgG, mg/dL				
<700	1 (1.1)	0	1 (0.8)	0.50
700–1600 ^a^	90 (98.9)	42 (100.0)	132 (99.2)	
>1600	0	0	0	
Mean (SD)	925.7 (131.5)	989.6 (94.7)	945.4 (124.5)	0.001
IgA, mg/dL				
<70	0	0	0	-
70–400 ^a^	91 (100.0)	42 (100.0)	133 (100.0)	
>400	0	0	0	
Mean (SD)	225.7 (32.9)	248.3 (26.3)	232.6 (32.6)	<0.001
IgE, mg/dL				
0~200 ^a^	91 (100.0)	42 (100.0)	133 (100.0)	-
>200	0	0	0	
Mean (SD)	123.6 (18.7)	135.7 (16.6)	127.3 (18.9)	<0.001

WBC, white blood cell. * chi-square and Kruskal–Wallis tests. ^a^ the normal reference value range.

**Table 4:** Spearman’s correlation coefficients of antimony exposure levels and immunological indicators of all study participants. (*N* = 133).

Immunological Indicators	Antimony in			
	Air	Blood	Urine	Hair
WBC	0.135	0.010	0.126	0.143
Lymphocyte	−0.104	−0.106	−0.121	−0.137
Monocyte	−0.117	−0.300 **	−0.175 *	−0.164
IgG	−0.260 *	−0.026	−0.157	−0.187 *
IgA	−0.366 **	−0.291 **	−0.355 **	−0.370 **
IgE	−0.236 *	−0.171 *	−0.175 *	−0.217 *

^a^ Spearman’s correlation coefficients. * *p* < 0.05. ** *p* < 0.001.
